# Supplementary material for: Brain mass explains prey size selection better than beak, gizzard and body size in a benthivorous duck species
Source: PLoS One. 2021 Mar 30;16(3):e0248615. doi: 10.1371/journal.pone.0248615 (PMC8009388; doi:10.1371/journal.pone.0248615)
Supplement: Attachment — Submitted filename: Response to Reviewers.docx [file pone.0248615.s002.docx]

PONE-D-20-17519
Brain size explains prey size selection better than beak, gizzard and body size in a benthivorous duck species
PLOS ONE

**12/15/2020**

**Dear Academic Editor Dr. Vitor Hugo Rodrigues Paiva,**

**Thank you very much for your email with comments on our paper. We are grateful for giving us the possibility to improve our manuscript.**

**We have read the comments carefully and amended the manuscript accordingly. Some of the remarks from the reviewers were critical but some were also constructive. We have improved the text and explained in more detail what we have done. Below we have given comments to the points raised. All our comments are given in bold text starting with ‘-‘.**

**Please find enclosed three documents: a revised version without track changes, a revised manuscript with track changes and a document explaining in detail how we have addressed all points raised.

Thank you once more for your editorial assistance.**

**Yours sincerely,**

**Karsten Laursen, on behalf of the authors**

[Note: HTML markup is below. Please do not edit.]

Reviewers' comments:

Reviewer's Responses to Questions

**Comments to the Author**

1. Is the manuscript technically sound, and do the data support the conclusions?

The manuscript must describe a technically sound piece of scientific research with data that supports the conclusions. Experiments must have been conducted rigorously, with appropriate controls, replication, and sample sizes. The conclusions must be drawn appropriately based on the data presented.

Reviewer #1: Yes

Reviewer #2: Partly

2. Has the statistical analysis been performed appropriately and rigorously?

Reviewer #1: Yes

Reviewer #2: I Don't Know

3. Have the authors made all data underlying the findings in their manuscript fully available?

The [PLOS Data policy](http://www.plosone.org/static/policies.action#sharing) requires authors to make all data underlying the findings described in their manuscript fully available without restriction, with rare exception (please refer to the Data Availability Statement in the manuscript PDF file). The data should be provided as part of the manuscript or its supporting information, or deposited to a public repository. For example, in addition to summary statistics, the data points behind means, medians and variance measures should be available. If there are restrictions on publicly sharing data—e.g. participant privacy or use of data from a third party—those must be specified.

Reviewer #1: Yes

Reviewer #2: Yes

4. Is the manuscript presented in an intelligible fashion and written in standard English?

PLOS ONE does not copyedit accepted manuscripts, so the language in submitted articles must be clear, correct, and unambiguous. Any typographical or grammatical errors should be corrected at revision, so please note any specific errors here.

Reviewer #1: Yes

Reviewer #2: Yes

5. Review Comments to the Author

Please use the space provided to explain your answers to the questions above. You may also include additional comments for the author, including concerns about dual publication, research ethics, or publication ethics. (Please upload your review as an attachment if it exceeds 20,000 characters)

Reviewer #1: Comments for authors
Major comments
1. Introduction could be more streamlined and trimmed
Firstly, authors should try to be more clear in the text about separating which traits may be key for driving prey size selection at inter- and intraspecific levels. This could be pertinent for their own results as well. Also, I think, that Introduction’s first paragraph could be much more condense. Especially, I doubt if so much information about beak-related prey selection is really needed. In any case, it would be also great if you could be more specific and mention in the introduction about how well (less controversial as of recently) brain size is related to cognitive abilities. Lastly, information on study species would feel more natural when coming after presenting main hypothesis (lines 99-101).

**-We now distinguish clearly between interspecific and intraspecific relations, see lines 56-61. In addition we have shortened the introduction, by deleting some of the text on beak-related selection, see lines 56-65 (in the original manuscript) and lines 55-61 in the revised version.**

**-Further, we have added some sentences about the debate of using brain size as a measurement of cognitive abilities, see lines 80-82.**

**-Lastly, there may be a misunderstanding, because information on the study species, the eider, is presented after (see lines 101-103) the hypothesis (see lines 91-92).**

2. Hypotheses
Authors may disagree, but as the hypothesis is written (99-101) it is not very well suited for the given study and more suitable for between seasonal comparison. Also, predictions are not well linked to hypothesis, but rather the results.

**-We agree, and have changed the hypothesis, see lines 91-92.**

3. Statistical analyses
Could you please clarify if in your analyses a relative rather than absolute brain size was used? Brain size is often corrected for body size as larger individuals tend to have larger heads. You seem to have used body mass in the same model, but it is not fully clear to me if body size was left in the model as covariate. Based on the result table it probably was and no model selection was done, but could you clarify that. Would you get a different outcome by using structural size estimate rather than body mass for scaling?

**-Sorry if the text could be confusing, but it is now clear from the text that all variables stayed in the model including body mass, see lines 162-163. The reason that we included body mass was two-fold. First that we wanted to include body mass because studies have shown that it was a variable that could determine prey size in accordance with i. e. beak size. Second, as the referee mentions brain size can depend on body size, by including body mass, we had reduced the possibility that a relationship between brain size and prey size was a trivial relationship. The reason for not including both body mass and also a structural variable as for example femur length, was that there is a significant relationship between body mass and femur length (t = 4.17; P < 0.0001). For these reasons, we selected body mass for the model.**

**-As suggested by the referee we try to include femur length as a structural parameter in the model, and found that there was some minor differences in the results depending on the prey type and size was analyzed. However, it did not change the original statistical relationships found. Thus, we did not include femur length in the model.**

Would there be a reason to suspect that YEAR needs to be included in the model (sampling areas, food availability etc.)? If not, would be nice to see explanation included in the statistical analysis description.

**-As suggested by the referee we have included YEAR in the model in the revised manuscript, see lines 156-159. The new analysis changed the results in two ways, (a) the importance of head volume in relation to prey size is now more clear, and (b) the relationship between shore crab and gizzard mass as shown in the original manuscript, was no longer statistical significant. Thus, the analysis of the relationship between the size of shore crab and area of webbed feet is no longer relevant. Thus, the paragraph is left out in the revised manuscript.**

Would using median for prey size rather than mean influence the results? Median value would perhaps be more appropriate in relation to „optimal“ selection as it would indicate that individual selected such item more frequently.

**-This is a good idea, and we have prepared the data. However, preliminary analyses showed highly significant correlation between mean prey size and median prey size. As an example the correlation between mean size and median size of one of the most abundant prey species, the blue mussel, showed a highly significant correlation between the two measurements (r^2^ = 0.98). For this reason, we assessed that including median size instead of mean size would not change or improve the results.**

As authors already have presence-absence data it could be interesting to see how prey diversity is related to brain size. Are bigger brained individuals going the diversification or the specialization route?

**-This is also a good suggestion, and we estimated the diversity using the Shannon Wiener index. However, there was no significant results (or even close to significance) when analyzing the diversity index in relation to the model (body mass, gizzard mass, beak volume, head volume and sex as explanatory variables). Due to this, we have not included these new results in the revised paper.**

4. Discussion
I failed to find any comments on possible sex-related differences in prey size selection. Also, what about beak volume, especially given the attention to this trait in the introduction. Please, place your results in relation to your hypothesis and introduction a bit more clearly than it is now.

**-Sorry for this forgetfulness. We have now included a paragraph on beak volume and sex-related differences. See lines 196-202.**

Minor comments:
Line 31: example of Calidris in the abstract is confusing. This specificity perhaps here is not needed.

**-We have deleted the example, and changed the first lines of the abstract, see lines 30-32.**

Lines 40-43 This is confusing sentence as it is not clear what were dependent variables in your study. As it is now written it seems that you looked at brain size vs other morphological traits.

**-The referee is right, the sentence was confusing. It is now corrected, see lines 38-41.**

Line 46: The last sentence of the abstract feels disconnected from previous description of results.

**-We have changed the sentence, see lines 46-48.**

Line 80 sentence is repetitive

**-The sentence is deleted.**

Line 83 Something is missing here. Perhaps a connector part on how cognitive abilities are increasingly recognized to be associated with brain size?

**-We have added some additional sentences, see lines 76-79.**

Line 95-98 a confusing sentence

**-It is a redundant sentence, and it have been deleted.**

Line 108 „..on the other“

**-The sentence is corrected, see line 101.**

Line 115 what is the trait „potential“? Should it be „potential for successful reproduction..“?

**-Correct, it is changed, see line 105-108.**

Line 119 strange confusing sentence that looks to be clipped from Methods section. Few lines down the same information is mentioned.

**-The sentence has been deleted.**

Line 126 Please mention also if the two sexes (plus juveniles) were roughly equally represented for different years.

**-The sex and age classes are now specified, see lines 122-124.**

Line 132 what do you mean for age here (juveniles vs sexually mature)? Or was the chronological age recorded?

**-This is now specified, see line 129.**

Line 136 Please, double-check if the provided reference (10:Zhang et al.) is correct for the formula. Zhang et al. did not measure head volume in their study. Also, check the formatting for the head volume formula.

**-The reference has been changed, see line 134.**

Line 139 More appropriate term for „head“ here would be „skull“. I agree with authors that it could make sense to measure dry brain weight. However, also in previous studies Jaatinen et al. 2019 wet (?) brain mass was measured with the same results. Perhaps mentioning why dry brain weight was more appropriate option (if that was so), how was it standardized and that there were infact no qualitative differences may be good (in case it is so).

**-‘Head’ has been changed to ‘skull’, see line 139.**

**-We have consistently used head size and brain size in our analyses because they are strongly positively correlated accounting for more than 75% of the variance (e.g. 16, 26, papers on eider and brain).**

Line 168 What statistical programme was used for data analyses?

**-We used SAS. 2012. JMP. Version 10.0.2. SAS Institute Inc, Cary, NC. See line 164 and 368.**

Line 202 check typo „ether“

**-The paragraph is now deleted.**

Lines 205-207 These are repetition from result section. Could be better to introduce a general pattern or main finding.

**-The referee is right. The sentence is now changed, see lines 187-189.**

Lines 216-222 This information actually would have been nice already in the introduction and not here.

**-The paragraphs has been moved to the introduction, see lines 109-115.**

Line 236 Apologies, but again a confusing sentence. Do you have in mind that you found extreme sizes or that eiders seemed to prefer larger than expected prey?

**-The sentence has been corrected, see lines 225-226.**

Line 238 what species was this done on? Also word „such as “ missing

**-The species is now added, see lines 226-229.**

Line 254 but you do not find that. If that is because lab vs natural condition then connection to the previous sentence should be clearer.

**-We have now clearly mentioned if we are writing about feeding under natural or experimental conditions, see lines 231 and 243-245.**

Line 255-258 very unclear sentence

**-The sentence was confusing, and it has been deleted.**

Line 259-267 I would try placing this para just before conclusions.

**-We have followed the advice and moved parts of this paragraph before the conclusion, see lines 250-254.**

Line 275 and 285 Would suggest to avoid „obviously“ and tone down.

**-The paragraph has been deleted.**

Reference list not formatted properly.

**-We have improved the reference list.**

Reviewer #2: The authors should change the title of the paper. For instance, their findings are not only positive relationship between brain volume and prey size but also positive relationship between the size of shore crabs and the area of web of feet.

**-The results of relationships between shore crabs and webbed feet have been deleted in the revised version. Thus, the title is not changed.**

The authors found positive relationship between head volume and brain mass (F = 39.21, df = 1,13, p < 0.0001, r2 = 0.73). But, can the head volume really be a proxy of the brain size? I agree that the head volume could be one of the indicators of the degree of brain development, though. Better idea would be that the authors use the volume of brain itself (or the size of brain domain specific for visual cognition) as the indicator of cognitive abilities.

**-The referee is right that data on the size of the brains for all 198 eiders would have been better for the analysis and probably giving a more precise result than using the size of the heads. However, we emphasize that the two variables are strongly positively correlated accounting for 75% of the variance (Jaatinen et al. 2019). Given that head size and brain size are tightly, positively correlated, it is unlikely that the conclusions would have changed if we had used one rather than the other.**

Introduction Line3-7: Accipritridae, Podicipedidae, and Fringillidae, for example, are all "Family" name of birds. So, the authors should write them in regular style, not in italic style.

**-The lines have been deleted.**

6. PLOS authors have the option to publish the peer review history of their article ([what does this mean?](https://journals.plos.org/plosone/s/editorial-and-peer-review-process#loc-peer-review-history)). If published, this will include your full peer review and any attached files.


If you choose “no”, your identity will remain anonymous but your review may still be made public.


**Do you want your identity to be public for this peer review?** For information about this choice, including consent withdrawal, please see our [Privacy Policy](https://www.plos.org/privacy-policy).

Reviewer #1: No

Reviewer #2: No

[NOTE: If reviewer comments were submitted as an attachment file, they will be attached to this email and accessible via the submission site. Please log into your account, locate the manuscript record, and check for the action link "View Attachments". If this link does not appear, there are no attachment files.]

While revising your submission, please upload your figure files to the Preflight Analysis and Conversion Engine (PACE) digital diagnostic tool, <https://pacev2.apexcovantage.com/>. PACE helps ensure that figures meet PLOS requirements. To use PACE, you must first register as a user. Registration is free. Then, login and navigate to the UPLOAD tab, where you will find detailed instructions on how to use the tool. If you encounter any issues or have any questions when using PACE, please email PLOS at [figures@plos.org](mailto:figures@plos.org). Please note that Supporting Information files do not need this step.

*In compliance with data protection regulations, you may request that we remove your personal registration details at any time.* [*(Remove my information/details)*](https://www.editorialmanager.com/pone/login.asp?a=r)*. Please contact the publication office if you have any questions.*
