# Supplementary material for: Brain mass explains prey size selection better than beak, gizzard and body size in a benthivorous duck species
Source: PLoS One. 2021 Mar 30;16(3):e0248615. doi: 10.1371/journal.pone.0248615 (PMC8009388; doi:10.1371/journal.pone.0248615)
Supplement: Attachment — Submitted filename: Response to Reviewer.docx [file pone.0248615.s003.docx]

PONE-D-20-17519R1
Brain size explains prey size selection better than beak, gizzard and body size in a benthivorous duck species
PLOS ONE

**Dear Academic Editor, Dr. Vitor Hugo Rodrigues Paiva,**

**Thank you very much for your email with comments on our paper. We are grateful for you giving us the possibility to improve our manuscript.**

**We have read the comments carefully and amended the manuscript accordingly. Some of the remarks from the reviewers were critical but also constructive. We have improved the text and explained in more detail what we have done. Below we have provided comments to the points raised. All our comments are given in bold font starting with ‘_’.**

**Please find enclosed three documents: a revised version without track changes, a revised manuscript with track changes and a document explaining in detail how we have addressed all points raised.**

**Thank you once more for your editorial assistance.**

**Yours sincerely,**

**Karsten Laursen, on behalf of the authors**

Dear Dr. Laursen,

Thank you for submitting your manuscript to PLOS ONE. After careful consideration, we feel that it has merit but does not fully meet PLOS ONE’s publication criteria as it currently stands. Therefore, we invite you to submit a revised version of the manuscript that addresses the points raised during the review process.

Please submit your revised manuscript by Mar 04 2021 11:59PM. If you will need more time than this to complete your revisions, please reply to this message or contact the journal office at [plosone@plos.org](mailto:plosone@plos.org). When you're ready to submit your revision, log on to <https://www.editorialmanager.com/pone/> and select the 'Submissions Needing Revision' folder to locate your manuscript file.

Please include the following items when submitting your revised manuscript:

- A rebuttal letter that responds to each point raised by the academic editor and reviewer(s). You should upload this letter as a separate file labeled 'Response to Reviewers'.
- A marked-up copy of your manuscript that highlights changes made to the original version. You should upload this as a separate file labeled 'Revised Manuscript with Track Changes'.
- An unmarked version of your revised paper without tracked changes. You should upload this as a separate file labeled 'Manuscript'.

If you would like to make changes to your financial disclosure, please include your updated statement in your cover letter. Guidelines for resubmitting your figure files are available below the reviewer comments at the end of this letter.

If applicable, we recommend that you deposit your laboratory protocols in protocols.io to enhance the reproducibility of your results. Protocols.io assigns your protocol its own identifier (DOI) so that it can be cited independently in the future. For instructions see: <http://journals.plos.org/plosone/s/submission-guidelines#loc-laboratory-protocols>

We look forward to receiving your revised manuscript.

Kind regards,

Vitor Hugo Rodrigues Paiva, Ph.D.
Academic Editor
PLOS ONE


[Note: HTML markup is below. Please do not edit.]

Reviewers' comments:

Reviewer's Responses to Questions

**Comments to the Author**

1. If the authors have adequately addressed your comments raised in a previous round of review and you feel that this manuscript is now acceptable for publication, you may indicate that here to bypass the “Comments to the Author” section, enter your conflict of interest statement in the “Confidential to Editor” section, and submit your "Accept" recommendation.

Reviewer #1: (No Response)

Reviewer #3: (No Response)

2. Is the manuscript technically sound, and do the data support the conclusions?

The manuscript must describe a technically sound piece of scientific research with data that supports the conclusions. Experiments must have been conducted rigorously, with appropriate controls, replication, and sample sizes. The conclusions must be drawn appropriately based on the data presented.

Reviewer #1: Yes

Reviewer #3: No

3. Has the statistical analysis been performed appropriately and rigorously?

Reviewer #1: Yes

Reviewer #3: Yes

4. Have the authors made all data underlying the findings in their manuscript fully available?

The [PLOS Data policy](http://www.plosone.org/static/policies.action#sharing) requires authors to make all data underlying the findings described in their manuscript fully available without restriction, with rare exception (please refer to the Data Availability Statement in the manuscript PDF file). The data should be provided as part of the manuscript or its supporting information, or deposited to a public repository. For example, in addition to summary statistics, the data points behind means, medians and variance measures should be available. If there are restrictions on publicly sharing data—e.g. participant privacy or use of data from a third party—those must be specified.

Reviewer #1: No

Reviewer #3: Yes

5. Is the manuscript presented in an intelligible fashion and written in standard English?

PLOS ONE does not copyedit accepted manuscripts, so the language in submitted articles must be clear, correct, and unambiguous. Any typographical or grammatical errors should be corrected at revision, so please note any specific errors here.

Reviewer #1: Yes

Reviewer #3: No

6. Review Comments to the Author

Please use the space provided to explain your answers to the questions above. You may also include additional comments for the author, including concerns about dual publication, research ethics, or publication ethics. (Please upload your review as an attachment if it exceeds 20,000 characters)

Reviewer #1: Response to authors:
I thank the authors for answering my questions and making changes to their analyses or/and text. The manuscript seems to be much improved. Yet, I would still have a couple of comments which authors may take into their consideration. Also, I would suggest authors to carefully go through their manuscript to improve style and fluency of the text.

_**Thank you for the comments, which we have taken into account.**
##############################################################


Minor comments:

L 42 „positively“

**_Corrected.**

L60 „differences“ not needed?

**_Deleted.**

L63 “by“ to „with“

**_Corrected.**

LL91-116. The whole paragraphs may still benefit from careful reading of the text. Part of the problem may be change in the tense starting at L96. If you do not want to change the place where study species is introduced, it could be more clearer if you wrote „....Body mass and sex should be included in such analyses as for some bird species these traits are known to have an effect on prey size selection (5,23).” Or similiar. Similar suggestion would be for the next sentence.

**_We have included the sentence suggested by the referee about body mass and sex, and changed the sentence accordingly. See lines 97-99.**

L 96 it could be debated if sex and body mass should be called anatomical structures.

**_The referee has a point here. The sentence is changed. See lines 96-97.**

L144 should be changed to „...we measured as the total (longest) length of the shell to the nearest XXmm.“ or similar

**_We have changed the description of assessing the size of the food items. See lines 154-157.**

L145-149 Sentence is difficult to understand. It could be broken into separate ones according to group.

**_The paragraph is changed. See lines 151-158.**

L149 Unclear. Perhaps „Size of broken items was approximated to the nearest 5mm using a size-appropriate reference from collection of intact prey samples“. Would that be what you mean to say?

**_Thank you for the suggestion. See l. 156.**

L150 „For each eider“ or „...individual..“, cause how many gizzards does one individual have?

**_Corrected.**

LL159-162 Sentence should be checked for clarity.

**_Corrected. The sentence was unclear. It is corrected. See lines 168-169.**

L164 Include, version for JMP if appropriate

**_We have added the version. See l. 173.**

L200 It is unclear then which sex took larger prey. In results it is stated that females take smaller prey, while in discussion it becomes no longer that clear.

**_Sex differences in prey size are interesting, but not an important point here. A reason to include sex in the statistical analysis is to reduce the variance in the data.**

L206-208 Sentence could be nicely split into two.

**_Thank you for the suggestion. See lines 227-230.**

L219 I would exclude word „Obviously“ or tone down this sentence. For human senses bivalves may be difficult to find, but we still know surprisingly little about bird senses including for instance olfaction.

**_We have done so.**

L241 If smaller fraction is lost, then your whole mean is shifted towards larger numbers and thus you are „overestimating“ rather than „underestimating“ mean prey size?

**_Sorry, of course the referee is right. It is corrected. See l. 264.**

LL 257-265 I imagine this part (Conclusions) should contain more general statements than the summary of results.

**_We have changed the wording. See lines 276-278.**

L413 (Fig 1 legend). Use of „Number (%) ...“ is confusing. Also, in the figure it self, perhaps spelling what is on y-axis would be more clear. Perhaps, "Proportion (%)..."

**_It is corrected. See l. 427.**

L424 Table 1.What are the numbers given in Table 1 and how are they related to those given in first lines of the results section. Are numbers provided in this table describe only intact items? If so, then it should be mentioned.

**_The referee has a point. It is now add in Tabel 1 that N is the number of eiders with a given prey type. Opposite to the first line in the Result Section, which is based on the total number of prey. See lines 442 and 177.**

Reviewer #3: Major comments
My main concern is that in its current state, this study shows a correlation of head size with prey item size, which may be just an artifact of the mechanical limit of ingesting big preys (i.e. only big birds with big mouths can ingest the big prey). This idea is supported by the fact that only the larger items show a correlation with head size. Small food items will not show a correlation because they are far from the mechanical limit, contrary to the expected if the eiders select for relative large preys. If the authors want to test if eating big prey items is a cognitively demanding task, a correlation of relative brain size (brain corrected by a measure of overall body size) with relative prey size would be a more suitable approach. If the hypothesis is true, individuals with relatively larger brains will ingest relatively larger items. Moreover, head size probably correlates with gape width too (it could have even a stronger relationship than head-brain size), so it is hard to distinguish between both effects. Therefore, the authors need to tone down their interpretations, this study is completely correlational, and statements such as “Brain size explains prey size selection” and “These novel results indicate that cognitive processes connected to brain size are involved in prey size selection by eiders” (L47-48), should be avoided.

**_Two important aspect were raised here by the referee.**

**_First, about the correlations. It is correct that if the overall size of the eiders was not included in the analyses, we would just get a trivial correlation showing that eiders with big heads took large prey. However, we had included body size (as body mass) in all analyses, to eliminate this effect.**

**_Second, the gape width is a good point raised by the referee. To be honest, we have not been thinking about the aspect of gape width. However, it sounds reasonable that the gape could set limits for the prey size taken, even that the hinge between the upper and lower mandible is flexible. We had an opportunity to examine the gape of 21 eiders sampled in February 2021. The mean (se) of the gape width was 44.4 (0.6) mm, the gape height 39.3 (0.9) mm. From our reference sample of intact benthos species, we measured length and width relations of blue mussels, razor clams, cockles, conch and shore crab. From our data file of diet, the largest blue mussel taken by the eider was 66 mm, for cockle 45 mm, for razor clam 148 mm, for conch 68 mm and for shore crab 55 mm. The corresponding width for the longest blue mussel is 28 mm, for cockle 42 mm, for razor clam 22 mm, for conch 40 mm and for shore crab 38 mm. The measurements for all food items are smaller than the mean width of the gape (44.4 mm). From this, we considered it unlikely that the dimensions of the gape should restrict intake of food items in eiders. We have included a short summary in the revised paper, as an alternative hypothesis. See lines 118-121, 158-159 and 194-206.**

Also, the authors use the term “size” quite often throughout the manuscript, but sometimes it actually has different meanings (e.g. volume, length, mass), which blurs the interpretation. For example, this paper used volume as a measure of beak size, which is different from the measure used in reference 9 (i.e. beak length: L62-63). Using the actual variables, when available, will facilitate the comprehension and transferability of the conclusions.

**_Sorry for using ‘size’ as a general expression for a dimension. It was done simply to make the reading more straightforward and easy. We have changed the wording accordingly. However, we will still use beak volume as a measurement for beak dimension, since in our opinion it makes the statistical analyses simpler (one variable instead of three variables).**

Minor comments
Abstract
L30 This opening statement is not quite true. Even though this was a common hypothesis in the literature, it does not hold in most studies. The authors do a good job in the introduction showing how this is not always true, therefore this statement should be toned down.

**_We have changed the sentence. See lines 30 and 55.**

L32 This paper did not use the total brain size. Then, the authors present the actual variable (L37), So I suggest deleting or editing this statement to reflect what is actually done.

**_We have skipped ‘total’ and changed ‘brain size’ to ‘brain mass’, which was actually measured. Brain mass is used throughout in the revised paper. See lines 32, 37, 38 and 47.**

L41 Hinia reticulata** (L140 too).

**_Sorry, this is now corrected, see lines 41 and 146.**

L45 “Strongly” is a bit subjective, your results show a significant effect, but it is not necessarily strong.

**_It is deleted. See l. 45.**

Introduction
L59-60 I think this idea describes reference 4 instead of reference 6, and maybe it needs to be rephrased… Diet explains a small fraction of the shape’s variation, instead of a small fraction of the diet explains variation. Right??

**_The referee is right. The sentence is changed. See l. 60-62.**

L62-63 Is reference 9 based on Calidris canutus or Calidris tenuirostris??? Please revise the reference

**It is correct. The species name has been changed. See l. 63.**

L69-73 Do eiders (or other birds) ever eat items as large as the gizzard?? Or this feedback mechanism would affect the number of preys (total volume) rather than the size of each individual item. For instance, the authors found 1299 items in 198 individuals (average 6.5 prey items). This might be important to discuss later.

**_There are some good point raised here by the referee.**

**_First, the prey size in relation to gizzard size. At some occasions, eiders have taken razor clams larger than the gizzard. One end of the clam was in the gizzard and the other end in the esophagus. The reason to take such a large prey could be that it represents a large amount of flesh and that it is soft shelled. We have added a sentence about these observations. See lines 247-249.**

**_Second, the question if the amount of food in the gizzard has a sort of feedback mechanism on selection on each item. This is an interesting question, which we have not considered, to be frank. However, the question cannot be answered directly, because we don’t know in which order the prey items in the gizzard have been taken. However, we can get an impression from our data. If there is a sort of feedback, we should expect few and large preys in the gizzards in contrast to many and small preys. The data reviles the opposite result, that there was a positive relationship between the number of preys and the mean prey size in the gizzards (r^2^ = 0.26) and for the maximum prey size (r^2^ = 0.22). Which does not support the assumption. Due to these points being marginal, we will not pursue this item further.**

L80 Does it refers to absolute and relative brain size, rather than “simply brain size”

**_This is now corrected for brain mass. See l. 81.**

L98 Head volume is a pretty rough approximation for brain size, authors need to acknowledge that and be careful with the interpretation. For instance, ~25% of the variation in brain size is not explained by head size (L174).

**_The referee is right. On the other hand, in most ecological studies only about 8% of the variation was explained (Oecologia 132, 492-500).**

Methods
L121 By convention latitudinal coordinates go before longitudinal coordinates. i.e. (55 ̊ 50’ N; 10 ̊ 20’ E)

**_Sorry, of course. It is corrected. See l. 125.**

L135-136 It is unclear to me why authors fitted an ellipsoid on a non-elliptical object (the beak) to get a measure of size (volume), if this a common practice or for comparative purposes, please cite. Wouldn’t be better to combine the three variables into a single one using a PCA to get a measure of “size”??

**_We used estimates of volumes when calculating the size of the head (other variables and prey (razor clam for example)). We did so because previous studies of brain size have been based on ellipsoids (2 radii of similar size and one that is larger) rather than circular volumes (3 radii of similar size). Proc. R. Soc. Long. B; (2002) 269:961-967. PLOS One (2020) 15 (9): e0236155.**

**_The use of Principal Component Analyses based on the correlation matrix from the log-transformed data, we still do not find any evidence consistent with the expectations suggested by the reviewer, and only one of the relationships reached statistical significance.**

L148-149 I do not understand how you are using this number later in your results, and why to divide it by four, please clarify. Is this a common practice? Please cite.

**_It is simply to get the number of crabs. They have four claws, and when we count four claws of the same size the eider have taken (at least) one crab. We have corrected the text. See line 154.**

L157-… by use of a multivariate GLM. This may help to clarify that all dependent variables are included in a single model.

**_Thank you, it is now added to the text. See l. 166.**

Results
L181-182 It should say a “significant positive effect” instead of a “high positive effect”.

**_Yes of course. It is corrected. See lines. 182-183.**

L183 What is “(0.0193)”?

**_It is the ‘se’ value. It is now corrected. See l. 192.**

Discussion
L192 I agree that, historically, beak morphology has been hypothesized to have a direct relationship with food item characteristics. This is particularly true when the beak is used to manipulate the food, like finches cracking strong seeds. However, several studies have shown that species that ingest the items whole (as is the case of the common eider), the item size is limited or correlated with gape width (e.g. Hulsman 1981, Wheelwright 1985, Saunders et al. 1995, Dehling et al. 2016). This should be included in the discussion.

**_We have included gab size in the revised manuscript. See lines 118-121, 158-159 and 194-206.**

L194-196 Perhaps rephrase this: “These results support previous findings that, in general, other morphological traits than beak size drives prey size selection [4,6]”

**_Thank you for this suggestion. The sentence has been used. See lines 216-217.**

L200 To me, it seems advantageous for both sexes to build up body condition during winter, not just for females. Authors may argue that females are under stronger pressure due to nesting. However, the results showed that females eat smaller prey items (L178-179).

**_Thank you for this suggestion. It is included. See lines 222-224.**

L219 I would suggest avoiding “obviously” on a scientific manuscript and tone down this claiming. Especially, if it is unknown how easily the eiders would localize the bivalves by the siphons.

**_The word has been deleted, and the text toned down. See lines 240-242.**

L225 “larger prey size than expected” Where this expectation come from? This does not seem to be a prediction of this study. Is this based on literature?? Please clarify.

**_The sentence has been changed. See l. 246.**

L239-241 If smaller items are digested quickly, your sample would be skewed to the larger items. Thus, you are likely overestimating prey size.

**_Yes of course. It has been changed. See l. 264.**

L246 “At the breeding grounds, eiders demonstrate complex behavior.”: Complexity is a tricky term and difficult to define. Also, this sentence is a bit vague and not directly linked to your results or the following ideas. I suggest modifying it.

**_The sentence have been deleted.**

L246-249 This paragraph seems disconnected. How is this paragraph linked to your project?

**_The paragraphs were written trying to give a full picture of eider ecology during winter and breeding. However, the referee is right, it is not connected to the study. Thus, it is deleted.**

L250-252 Shouldn't this state something like “seasonal variation in resources and conditions” instead of “the studies”? Perhaps a quick recapitulation of the main evidence would clarify how do you get to this idea.

**_Thank you for the suggestion. The sentence has been changed. See lines 269-271.**

L263-265 These results showed that individuals with large heads eat larger prey items, and in general, individuals with larger heads do also have larger brains. However, this pattern could be easily explained by a size constrain on prey size-gape width.

**_Gape size has been included in the study. See lines 118-121, 158-159 and 194-206.**

**_The Conclusion Section has been shortened. See lines 276-279.**

In general, the manuscript needs to be carefully read and edited, for grammatical and punctuation errors. For example (but not limited to)
L44 missing a period
L61 missing a comma after “Within species”
L183 missing a closing parenthesis
L191 missing a comma
L203 double tab

**_Thank you for the advice and for the corrections.**

7. PLOS authors have the option to publish the peer review history of their article ([what does this mean?](https://journals.plos.org/plosone/s/editorial-and-peer-review-process#loc-peer-review-history)). If published, this will include your full peer review and any attached files.


If you choose “no”, your identity will remain anonymous but your review may still be made public.


**Do you want your identity to be public for this peer review?** For information about this choice, including consent withdrawal, please see our [Privacy Policy](https://www.plos.org/privacy-policy).

Reviewer #1: No

Reviewer #3: No

[NOTE: If reviewer comments were submitted as an attachment file, they will be attached to this email and accessible via the submission site. Please log into your account, locate the manuscript record, and check for the action link "View Attachments". If this link does not appear, there are no attachment files.]

While revising your submission, please upload your figure files to the Preflight Analysis and Conversion Engine (PACE) digital diagnostic tool, <https://pacev2.apexcovantage.com/>. PACE helps ensure that figures meet PLOS requirements. To use PACE, you must first register as a user. Registration is free. Then, login and navigate to the UPLOAD tab, where you will find detailed instructions on how to use the tool. If you encounter any issues or have any questions when using PACE, please email PLOS at [figures@plos.org](mailto:figures@plos.org). Please note that Supporting Information files do not need this step.

*In compliance with data protection regulations, you may request that we remove your personal registration details at any time.* [*(Remove my information/details)*](https://www.editorialmanager.com/pone/login.asp?a=r)*. Please contact the publication office if you have any questions.*
